# Stepping Stones Triple P: the importance of putting the findings into context – a response to Tellegen and Sofronoff

**DOI:** 10.1186/s12916-015-0289-4

**Published:** 2015-02-19

**Authors:** Sijmen A Reijneveld, Marijke Kleefman, Daniëlle EMC Jansen

**Affiliations:** Department of Health Sciences, University Medical Center Groningen, University of Groningen, PO Box 196, Groningen, 9700 AD The Netherlands; Department of Sociology and Interuniversity Center for Social Science Theory and Methodology (ICS), University of Groningen, Grote Rozenstraat 31, Groningen, 9712 TG The Netherlands

**Keywords:** Borderline to mild intellectual disability, Children, CONSORT, Parenting support, Psychosocial problems, Randomized controlled trial

## Abstract

Recently, we reported the findings of a randomized controlled trial on the effectiveness of Stepping Stones Triple P (SSTP) compared to Care as Usual (CAU), in *BMC Medicine*. The study involved parents of 209 children with Borderline to Mild Intellectual Disability (BMID), included following a school-based assessment of psychosocial problems. We found that SSTP had some short-term advantages over CAU, i.e., a reduction of parenting stress and of teacher-reported psychosocial problems, but no long-term advantages, at 6 months after the intervention. Tellegen and Sofronoff criticized that we included a limited amount of studies on the effectiveness of SSTP, and that the interpretation of our findings was inadequate. Regarding available evidence, we confined our summary to published high-quality RCTs regarding individual SSTP on level 4 – our RCT concerned that type of SSTP. Consequently, many studies were excluded but in a very adequate way. Regarding interpretation, Tellegen and Sofronoff criticized that we compared SSTP with CAU, but seem to be unware that this is consonant with current guidelines. Moreover, they noted that 49% of the parents who started SSTP followed less than half of the intended number of sessions. However, our findings on those who completed SSTP showed no more advantages of SSTP in the long term than CAU. We therefore stick to our conclusion that SSTP has some advantages in the short term compared to CAU, but not in the long term. The major burden of psychosocial problems in children with BMID prompts for further improvements.

Please see related articles: http://www.biomedcentral.com/1741-7015/12/191 and http://www.biomedcentral.com/1741-7015/13/25

## Background

In a recent paper in *BMC Medicine*, we reported the findings of a randomized controlled trial (RCT) on the effectiveness of Stepping Stones Triple P (SSTP) compared to Care as Usual (CAU) [1]. We appreciate the comments of Tellegen and Sofronoff on this paper [2]. A public debate on the quality of research is the best way to optimize research on what really works for children with psychosocial problems. This is indeed essential to make progress in solving the societal challenge of a high burden of psychosocial problems among children, and holds even more for children with Borderline to Mild Intellectual Disability (BMID) [3,4].

Our findings were based on an RCT, performed and reported following the CONSORT criteria for RCTs [5], and following the design as published in advance in a study protocol [6]. This RCT included 209 parents, 111 of whom were randomized to SSTP and 98 to CAU. In doing so, we followed the same procedure as in a previously published RCT on primary care Triple P (Triple P level 3), that showed no advantages of that variant of Triple P, compared to CAU [7,8]. Our findings were, first, that SSTP had some short-term advantages over CAU, but no long term advantages, i.e., at 6 months after the intervention [1]. Short-term advantages concerned parent-reported parenting stress and teacher-reported child psychosocial problems. Second, we found that 49% of parents dropped before completing at least five sessions, i.e., before half of SSTP [1].

## Summary of evidence and interpretation

Tellegen and Sofronoff commented on two issues in particular: the fact that we included a limited amount of studies on the effectiveness of SSTP, and that we did not adequately interpreted our findings. We will respond to these issues consecutively. First, regarding the issue of the existing evidence, we confined the summary of studies in the introduction of our paper to published RCTs regarding individual SSTP on level 4, as our RCT concerned that type of SSTP. As a result, we only cited a limited number of studies. The review of Tellegen and Sanders [9] comprised mostly studies of poor quality. In our paper, we noted some problems associated with the poor quality of those studies, and refer to the extensive discussion of Wilson et al. on that topic [1,10], which had already been published shortly before the submission for publication of Tellegen and Sanders paper [9]. We thus adequately included only studies that fully met criteria regarding having a high-quality design.

Second, regarding our interpretation of the RCT outcomes, Tellegen and Sofronoff criticize two issues. First, they indicate that examination of the mean scores on the measures shows that the lack of long-term effects might be explained by outcomes in the CAU group continuing to improve over the follow-up period. They further indicate that, in contrast, the parents in the SSTP group maintained improvements that were seen at short-term, and hence both groups showed some improvement. These notions are fully consonant with our interpretation of the findings: SSTP may improve the outcomes of children and families somewhat, but in the long term, it does not do that in a better way than the routine Dutch CAU for children with psychosocial problems. SSTP thus has no advantages compared to CAU in the long term.

Tellegen and Sofronoff object to the comparison that we made in our RCT of SSTP with CAU. However, they seem not to unaware that this feature of our RCT reflects the gold standard of research on effectiveness, the CONSORT guideline: a new intervention should be compared to the best care that is available [5]. Withholding that care would lead to an invalid measurement of efficacy, and would also go against ethical guidelines: the control group cannot be withheld the routine care. The new intervention should then simply prove that it is better than routine care – which seems not to be the case in the longer run for SSTP.

As a second criticism regarding our interpretation, Tellegen and Sofronoff comment on the fact that, in our RCT, 49% of all parents followed less than half of the intended number of sessions, i.e., they dropped out before completing five sessions. That is a high percentage indeed, and it certainly requires additional study on its reasons. This drop out could be due to many of the included parents having perceived a need for parenting support, and thus provided consent to participate; however, in due course, they decided that SSTP did not adequately meet that need. More importantly, we did not find advantages in the long term compared to CAU for the 51% (i.e., 57) of parents who did complete SSTP adequately, nor did we find more advantages for these 57 parents in the short-term than we did for the full SSTP group. We will address the issue of a high drop-out in additional analyses because of its major importance.

## Conclusions

In short, we highly appreciate the willingness of Tellegen and Sofronoff to enter the public debate on the effectiveness of SSTP, but we disagree on their critical reflection regarding the potential weaknesses of our RCT. This RCT, and the previously reported RCT on Primary Care Triple [8,10] have circumvented many of the weaknesses that have been noted regarding the previous research on the effectiveness of Triple P variants [1,10]. It concerned an adequately powered RCT which, compared with CAU (and not with a waiting list condition without care), used questionnaires that were not specifically developed for or used in the SSTP intervention, had an independent data collection process, i.e., parents were asked to complete questionnaires in the absence of the health care professional who was carrying out the intervention, and had a very high retention of participants in all measurements.

Our conclusion is that SSTP has some advantages in the short term compared to CAU, but not in the long term, at least in the Dutch setting. This requires further study, given the major burden of psychosocial problems in children with BMID, as well as for their parents and society.

## Abbreviations

BMID: Borderline to Mild Intellectual Disability
CAU: Care as Usual
RCT: Randomized controlled trial
SSTP: Stepping Stones Triple P
